# Occipital abscess associated with cholesteatoma: a case illustrating possible infectious spread

**DOI:** 10.1016/j.bjorl.2026.101825

**Published:** 2026-05-19

**Authors:** Kanu Lal Saha, Bishwajit Bhowmik

**Affiliations:** a Bangladesh Medical University, Department of Otolaryngology-Head and Neck Surgery, Shahbag, Dhaka, Bangladesh; b Bangladesh Medical University, Department of Radiology and Imaging, Shahbag, Dhaka, Bangladesh

## Abstract

• First reported occipital subperiosteal abscess secondary to pediatric cholesteatoma.
• Infection spread via thrombosed sigmoid sinus and patent mastoid emissary vein.
• CT and contrast MRI delineated sinus thrombosis, tract, and occipital abscesses.
• Modified radical mastoidectomy with abscess drainage achieved durable cure.

## Introduction

Extracranial complications of suppurative otitis media include facial nerve paralysis (the most common), followed by subperiosteal abscess, often associated with cholesteatoma, and labyrinthitis. A subperiosteal abscess may arise from direct erosion of the cortical bone or via transmastoid spread of infection through cribriform vessels.^1^^,^^2^ While subperiosteal abscesses typically present in the postauricular region over the mastoid bone, they can occasionally occur in atypical locations. Notably, the first case of an occipital abscess secondary to acute mastoiditis was reported in a nine-year-old boy.^3^ Here, we present a rare case of an occipital abscess resulting from cholesteatoma in a 12-year-old child.

## Case report

**Fig. 1:**
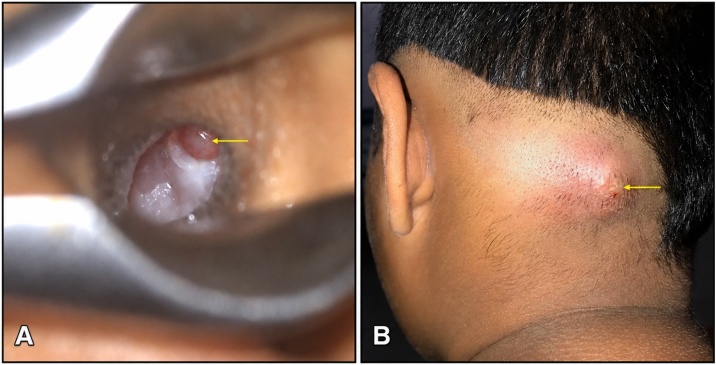
(A) Otomicroscopy demonstrated an attic (epitympanic) perforation with whitish desquamated debris, partially obscured by granulation tissue (yellow arrow). (B) Pre-operative photograph showing an abscess in the occipital region (yellow arrow).

**Fig. 2:**
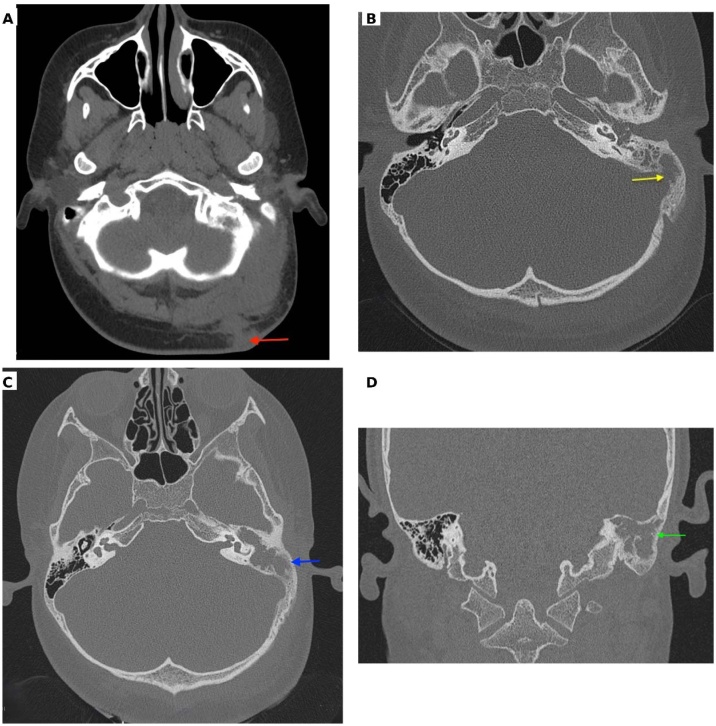
(A) Axial CT scan showing a soft tissue swelling at occipital region (red arrow). (B) Axial CT scan (bone window) showing lesion in the mastoid bone with erosion of the sinus plate (yellow arrow). (C) Axial CT scan showing intact cortical bone (blue arrow). (D) Coronal CT scan showing intact cortical bone (green arrow).

**Fig. 3:**
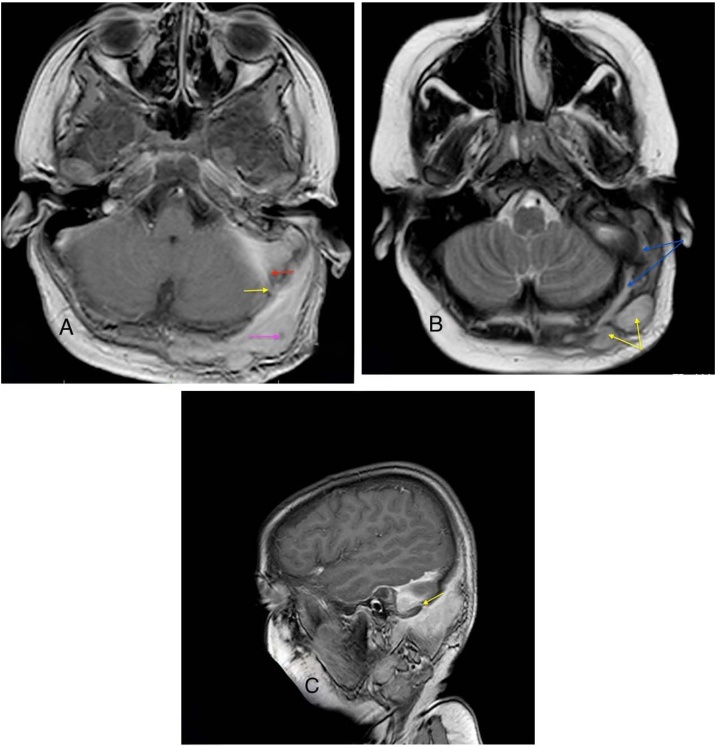
(A) T1WI MRI post contrast axial showing sigmoid sinus thrombosis (red arrow) and scalp abscess (pink arrow) connected with emissary vein (yellow arrow). (B) T2 WI axial MRI showing long sinus tract extended from the mastoid cavity to the soft tissue of left occipital region (blue arrow) connecting multiple abscess (yellow arrow). (C) Post contrast Sagittal T1WI MRI showing communication between infected sigmoid sinus and scalp lesion through emissary vein (yellow arrow) across the mastoid foramen.

**Fig. 4:**
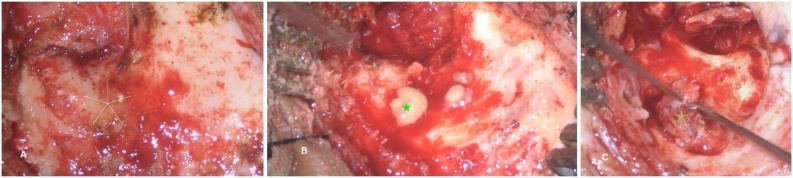
(A) Intact cortical bone (white star). (B) Erupting pus after removal of cortical bone (green star). (C) Decompressing the thrombosed lateral sinus (yellow star).

A 12-year-old boy presented with purulent, foul-smelling discharge and hearing impairment in his left ear for the last 4- to 5-years, along with painful swelling in the left occipital region for the past 15-days. He has received treatment at a district hospital that included intravenous antibiotics, ear drops, and analgesics. After achieving relief from the acute symptoms, he was referred to a tertiary center for definitive treatment. On otomicroscopic examination, there was an attic (epitympanic) perforation in the pars flaccida with whitish desquamated debris suggestive of cholesteatoma, partially covered by granulation tissue (Fig. 1A). Erosion of the scutum was also noted. The pars tensa was intact but congested and bulging. A soft, red, tender swelling measuring approximately 4 × 3.5 cm was noted in the left occipital region, suggesting abscess formation (Fig. 1B). Complete blood count revealed 80% neutrophil, ESR = 65 mm/hr and Hb% = 12.30 g/dL. Computed Tomography (CT) of the temporal bone without contrast demonstrated a separate soft-tissue swelling in the occipital region (Fig. 2A), along with soft-tissue density in the left middle ear cleft associated with erosion of the scutum and sigmoid sinus plate (Fig. 2B). However, the mastoid cortical bone remained intact (Fig. 2C‒D). Contrast-enhanced T1-weighted Magnetic Resonance Imaging (MRI) axial view revealed a filling defect (thrombus) in the left sigmoid sinus, connected via the emissary vein to an enhancing soft tissue lesion in the left occipital region with central necrosis, indicative of an abscess (Fig. 3A). T2 WI MRI axial view showed long sinus tract extended from the mastoid cavity to the soft tissue of left occipital region connecting multiple abscess (Fig. 3B) and post contrast sagittal T1WI MRI showed communication between infected sigmoid sinus and scalp lesion through emissary vein across the mastoid foramen (Fig. 3C). The patient underwent a left modified radical mastoidectomy. Intraoperative findings included cholesteatoma and granulation tissue occupying the epitympanum, sinus tympani, oval window, round window, aditus ad antrum, and mastoid antrum. The malleus head and incus were eroded, while the stapes superstructure remained intact and mobile A perisinus abscess with granulation tissue occluding the sigmoid sinus was found. The cortical mastoid bone was intact, but pus erupted from the lateral sinus wall and mastoid antrum upon initiation of mastoidectomy (Fig. 4A‒B). A type III tympanoplasty was performed after complete disease clearance. The thrombosed lateral sinus was decompressed, and the perisinus abscess and granulation tissue were extensively removed (Fig. 4C). A separate incision was made to drain the occipital abscess. Following incision and drainage of the abscess, a povidone-iodine-soaked gauze pack was placed and removed after 48 hours. Thereafter, regular dressing was continued using EUSOL and povidone-iodine solution until complete wound healing. The skin gap healed by secondary intention with scar formation, without formal suturing. The mastoid cavity was packed with large pieces of gelfoam, and the meatoplasty area was packed with a medicated sterile gauze, Sofratulle (Framycetin Sulfate). After suture removal on postoperative day 7, the gauze was removed, and topical ciprofloxacin-dexamethasone ear drops along with 2% acetic acid drops were started for 3-weeks. Subsequently, only 2% acetic acid drops were continued until the cavity healed. During follow-up, the cavity was suction cleaned, and debris and granulation tissue were removed as needed.

**Fig. 5:**
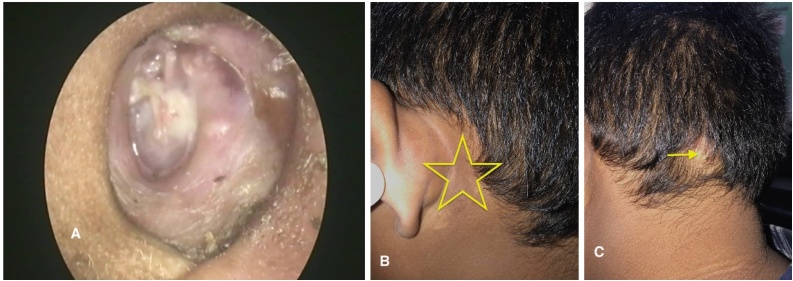
(A) Well-epithelialized dry mastoid cavity with intact neo tympanic membrane. (B) Healed postauricular region (yellow star). (C) Healed occipital abscess site (yellow arrow).

A dry, well-epithelialized mastoid cavity with a new, intact tympanic membrane was achieved within 3- or 4-months (Fig. 5A). The post aural area and occipital abscess site have completely healed (Fig. 5 B‒C). The patient has remained asymptomatic for 24-months following the surgery.

Patient’s parent provided the consent for the publication of this case report.

## Discussion

A subperiosteal abscess refers to the accumulation of purulent material within the potential space between the cortical bone and the overlying periosteum, most commonly arising as a complication of mastoiditis secondary to acute or chronic otitis media. The most frequently encountered form is the postauricular (mastoid) subperiosteal abscess. Less common sites include the zygomatic region (zygomatic abscess), Luc’s abscess, and Citelli’s abscess. The postauricular subperiosteal abscess over the lateral surface of the mastoid cortex commonly develops in children due to the transmastoid spread of infection through cribriform vessels following acute mastoiditis.^1^ In contrast, older children and adults are more prone to subperiosteal abscess formation caused by the direct erosion of cortical bone by cholesteatoma.^1^^,^^2^ Additionally, a subperiosteal abscess at the site of the mastoid emissary vein may also occur due to retrograde thrombosis or venous spread of infection from secondary to lateral sinus thrombosis.^4^ The classic presentation of a subperiosteal abscess includes painful postauricular swelling, obliteration of the postauricular groove, and forward displacement of the auricle. Although subperiosteal abscesses usually present over the mastoid region, atypical locations have occasionally been reported. The first documented case of an occipital abscess secondary to acute mastoiditis was described in a nine-year-old child with associated subdural abscess, extradural abscess, and lateral sinus thrombosis.^3^ In the present case of epitympanic cholesteatoma, the abscess was atypically located in the occipital region, with a clinically normal postauricular area. In the previously reported case, contrast-enhanced CT performed preoperatively demonstrated extradural, subdural, and occipital abscesses, which were confirmed intraoperatively; however, lateral sinus thrombosis was detected only on postoperative contrast-enhanced MRI.^5^

In our case, preoperative CT imaging and intraoperative findings confirmed an occipital abscess despite an intact mastoid cortex. Contrast-enhanced MRI plays a crucial role in the diagnosis of lateral sinus thrombosis and associated intracranial complications of mastoiditis.^4^^,^^5^ In current study, T2-weighted and contrast-enhanced T1-weighted MRI clearly demonstrated intraluminal thrombus, surrounding vascular involvement, and a dilated emissary vein connecting the lateral sinus to the occipital abscess. This suggests a direct venous pathway for infection spread from the mastoid cavity to the subperiosteal occipital region, resulting in multiple abscess formations.

Therefore, patients with cholesteatoma presenting with atypical abscess locations should be evaluated using both CT and contrast-enhanced MRI to ensure accurate diagnosis and appropriate surgical planning.

To the best of our knowledge, an occipital abscess associated with cholesteatoma has not been previously reported in the literature.

## Conclusion

Occipital abscess formation may occur through the spread of infection from a thrombosed lateral sinus via a patent mastoid emissary vein. In cases of cholesteatoma with unusual abscess locations or atypical clinical presentations, combined use of contrast-enhanced MRI and CT scanning is strongly recommended for precise diagnosis and optimal management.

## ORCID ID

Kanu Lal Saha: 0000-0002-5704-7518

Bishwajit Bhowmik: 0000-0001-8292-0633

## Authors’ contributions

Kanu Lal Saha: Conceptualization; data curation; writing- original draft; writing-review and editing.

Bishwajit Bhowmik: Conceptualization; supervision; writing-review and editing.

## Informed consent

Written informed consent was obtained from the patient’s parent who agreed to take part in the study.

## Ethical statement

All ethical considerations were addressed during the study design and implementation. Approval from the Bangladesh Medical University Institutional Review Board was not required for the publication of a case report.

## Funding

This clinical report did not receive any specific grant from any funding agency.

## Data availability

The authors declare that all data are available in repository.

## Declaration of competing interest

The authors declare no conflicts of interest.
